# The Rewards and Stresses of Teaching for Infant and Toddler Educators

**DOI:** 10.1007/s10643-026-02123-w

**Published:** 2026-04-07

**Authors:** Holly E. Brophy-Herb, Salma El Saedy, Ann Stacks, Carla Caringi, Chi-Fang Tseng, Claire D. Vallotton, Jessica L. Borelli

**Affiliations:** 1 Department of Human Development and Family Studies, Michigan State University, 552 W. Circle Drive, East Lansing, MI 48824, USA; 2 Merrill-Palmer Skillman Institute, Wayne State University, Detroit, MI, USA; 3 School of Social Ecology, University of California-Irvine, Irvine, CA, USA

**Keywords:** Infant and toddler educators, Early childhood education, Positive psychology, Relationships, Stress

## Abstract

Emerging work on educator well-being highlights relational well-being, a new construct reflecting, in part, the quality of educators’ workplace relationships, as potentially central for supporting educators. Relational well-being is understudied in the context of educators’ stressors, as are educators’ experiences in the relationships central to their work – those with children, families, colleagues, supervisors - which comprise their relational well-being. This qualitative study sought to advance our understanding of relational experiences and their impacts. Interviews were conducted with 22 infant/toddler educators from a Midwestern, U.S. state, most of whom worked in Early Head Start programs. Thematic analyses confirmed that educators’ experiences are simultaneously stressful and rewarding. Challenging and positive feelings coexist and are often inseparable, suggesting the need to consider both dimensions to better understand well-being. Novel findings included: 1) the framing of educators’ stressful and positive relational experiences as intrapersonal and interpersonal and the intersection of these dimensions; 2) educators’ explanations of the nuanced, challenging interactions with “upper” administrators with whom they had less frequent contact, and 3) educators’ intentional use of positive intrapersonal and interpersonal experiences to cope with stressors, and reduce the impacts of stressors on their interactions with children. Findings underscore educators’ intentional centering of children in their work and highlight educators’ use of rewarding experiences to sustain them and support responsive caregiving. The intentional use of rewarding experiences as a buffer may illustrate one path through which positive feelings relate to well-being. However, while this strategy was protective for children, it represented another layer of stress for educators.

Infant and toddler educators actively work to build warm, secure relationships that foster children’s development, despite reporting high rates of stress and burnout (Bryant et al., 2023; National Head Start Association, 2022; Russell, 2022). Work-related stress can increase turnover (Bryant et al., 2023; Carson et al., 2017; Kwon et al., 2025) and reduce classroom quality (Buettner et al., 2016; Jeon et al., 2019), both of which predict poorer child outcomes (Choi et al., 2019; Grant et al., 2019). In the last decade, much work has addressed how to understand how educators’ stress and well-being directly and indirectly impact children (Dreer, 2023; Jeon et al., 2022, 2024; Johnson et al., 2021) and how interventions have sought to reduce teacher stress and support responsive care (Avola et al., 2025). Understanding the nature of educators’ experiences is critical in identifying ways to better support educators in their profession. While professional development can buffer the impact of stress on classroom quality (Sandilos et al., 2020), effect sizes for professional development efforts aimed at reducing stress and burnout are generally small to moderate (Kestian, 2020), underscoring the need to better understand educators’ experiences of stress and well-being.

Interest in educators’ well-being has increased rapidly in recent years. In fact, a recent review found that more than half of the studies on defining educator well-being have occurred since 2019 (Jeon et al., 2026). Jeon and colleagues from the Early Head Start/Head Start Workforce Well-Being Consortium define well-being as a multi-component construct that includes both psychological and professional well-being, along with physical health and health behaviors. Results from their recent systematic review (2026) suggested three points relevant to the current study. First, studies of educator well-being have largely focused on educators’ depressive symptoms, personal stress, anxiety, and general mental health, with very little attention to more positive elements of well-being, such as psychological capital (e.g., optimism, hope). Similarly, few studies have included an examination of positive workplace components of well-being, including the characteristics of supportive relationships with administrators (e.g., administrator recognition). Third, Jeon and colleagues explain that educator well-being is placed in the context of “relational well-being” (p. 200), a newly defined construct they present that includes “relationships with children, families, co-workers, personal relationships, interpersonal skills, and communication skills” (p. 201). As far as we know, the construct of relational well-being first appears in the most recent work from Jeon and colleagues. Hence, despite its promise as a construct, relational well-being has not yet been well-studied, nor have the relational experiences in the workplace that comprise relational well-being. We specifically focus on workplace experiences. By relational experiences at work, we mean the qualities that characterize educators’ relationships and interactions with children, families, colleagues, and supervisors. This is important because early childhood programs are complex systems comprised of multiple relationships, including those among educators with children and families, with colleagues, and with their supervisors, all of which may be sources of both support and stress simultaneously (Beltman et al., 2020).

Jeon and colleagues’ work resonates with our own experiences listening to educators who participated in our professional development and evaluation study focusing on enhancing educators’ relationships with children and families and reducing educators’ stress (Stacks et al., 2024, Barron et al., 2023). In this work, educators suggested that workplace relationships were highly influential in their experiences of their work, including their work-related stress. The findings from that work led us to conduct this follow-up study to understand sources of stress, including “relational” sources (i.e., stress experiences in various workplace relationships), why they may or may not impact responsive caregiving, and what experiences help educators remain in the work despite the stress. Throughout this paper, we use the terms “relational” and “relationship-based” to characterize the nature of educators’ workplace experiences as largely embedded in relationships, primarily with children and families, colleagues, and administrators.

## Relationships with Children and Families

Much work on teacher stress and well-being to date has focused on two streams. First, Jeon et al.’s recent review (2026) showed that more than 60% of studies on educator well-being highlighted depressive symptoms, personal stress, general mental health, and anxiety as indicators of well-being quality. Second, research has focused heavily on correlates of educators’ stress, such as educators’ characteristics, children’s behaviors, and classroom characteristics (e.g., Clayback & Williford, 2022). While these streams of work are very valuable, they do not fully consider the positive experiences and rewards that draw early childhood professionals to the work and support their retention in the field. For example, early childhood educators report enjoying their work with the children in their classrooms, highly valuing the relationships they build with children and families, and feeling a sense of loyalty and commitment to them (Corbin et al., 2019; Hamel et al., 2025). These are prime examples of interpersonal experiences that educators have in their work. These interpersonal experiences with children and families are described as rewarding or fulfilling and may even be protective against the challenging aspects of this profession.

However, these relationships can also cause stress. Educators who experience conflictual relationships with children experience greater stress (Corbin et al., 2019). Even in the context of positive relationships with children, educators may encounter challenges, particularly regarding children’s behaviors they may find difficult (Clayback & Williford, 2022), or the greater emotional and physical dependence that infants and toddlers have on educators, compared to preschoolers. Moreover, infant and toddler educators, particularly, may experience relationships with families in intense ways, including parallels to coparenting or cocaring (Lang et al., 2016). Navigating families’ expectations and concerns, the nuances of communication, and differing cultural perspectives, for example, may present challenges for educators (Murphy et al., 2021). Recent research highlights work with children and families as rewarding but also challenging (e.g., Hamel et al., 2025). These relational complexities underscore the need to further understand the qualities and characteristics of educators’ experiences in their relationships with children and families (i.e., “relational” experiences).

### Colleague and Co-Teacher Relationships

Relationships with colleagues contribute to the climate in which educators work. For example, the co-teacher relationship is a particularly influential one, and unique to early childhood education (ECE), where co-teaching is common. Co-teacher relationships can be a source of support (Tebben et al., 2021) and are an important element in an ECE program’s climate (Hewett & La Paro, 2020). The quality of collegial relationships can impact educators’ job satisfaction and intention to stay (Kim, 2024; Wells, 2015) and predict lower risk of turnover (Bryant et al., 2023; McMullen et al., 2020; Ng et al., 2023; Yeh & Lo, 2024).

Early childhood educators generally report positive relationships with their co-educators and colleagues, characterized by helping behaviors (e.g., sharing resources and materials, problem-solving together), social support, and socializing together (Hewett & La Paro, 2020). Similarly, experiencing a sense of community and collegial support is related to educators’ satisfaction and commitment to their profession (McGinty et al., 2008). Alternatively, friction in these relationships may serve as an ongoing stressor for educators. Many studies of these relationships have used quantitative scales to assess collegial relationships, which provide a broader view into the nature of these relationships. In the current study, we seek to complement existing quantitative work and contribute to the concept of relational well-being with a more nuanced examination of infant and toddler educators’ relational experiences with their colleagues.

### Relationships with Administrators

Early in our work, infant and toddler educators told us that we needed to include administrators in relationship-based support for educators’ practices and well-being, eventually leading to the creation of joint educator-administrator materials (Caringi et al., 2025). In parallel, a growing literature links workplace climate (Lei et al., 2024), including positive teacher-administrator relationships, with early childhood educator well-being and retention (e.g., Vicente et al., 2025). Specifically, positive views of the workplace, including positive relationships with the administration, promote retention (Bull et al., 2024; Jones et al., 2020), job satisfaction (Xia et al., 2024), and feelings of professional efficacy (Jones et al., 2020; Waller, 2021).

There are various definitions of administrative support for educators. Many of these definitions include relationship-based, interpersonal aspects, such as administrators’ supportiveness, encouragement, clear communication of expectations (Conley & You, 2017), and their appreciation of educators and provision of resources (Kim, 2024). Although educator-administrator relationships may be intended to provide support, educators and administrators can have different perceptions of the quality of the program climate (Jorde-Bloom, 1988). That difference in perspective may in itself be stressful for educators. Further, administrators can contribute directly to educators’ stress when they devalue, micromanage, or undermine educators’ work with children and families (Kim, 2024).

Educators and administrators may also have different perceptions of what “support” entails (Kim, 2024). For example, Wiltshire (2024) found that early childhood educators found large group meetings less supportive, and they wished for “on-going and collaborative conversation” (pg. 663). This foundational body of work elucidates the complexity in relationships and some contradictions in experiences of educators and their administrators, and collectively, indicates that additional research is needed to better understand educators’ experiences of relationships with administrators. In the current study, we probed educators’ thoughts about what they wish administrators understood about the joys and complexities of their work with the intention to better understand the intricacies of educator-administrator relationships relative to educators’ daily experiences.

### Current Study

In summary, early childcare and education involves significant immersion in multiple relationships (McGinty et al., 2008; Zinsser & Curby, 2014). The complex experiences of ECE work calls for additional research to understand how educators experience and manage relational stressors and the support they need to provide responsive care. Using a relationship-based (or relational) framework to study educators’ stress and well-being reflects the recognition that educators’ well-being is shaped by the quality of their relationships, including those with children and families, colleagues, and administrators. Through a series of qualitative interview questions, this study probed infant and toddler educators’ relational experiences, including both stressors and experiences that help educators manage stress and remain in the field. We pursued the following research questions: 1) what are educators’ experiences of relational stress as well as experiences that sustain them, 2) how does relational stress impact educators’ responsive caregiving practices, and 3) how do educators mitigate the impact of stress on their responsive classroom practices?

## Method

### Participants

Twenty-two center-based infant and toddler female educators (54.5% (*n* = 12) Black, 18.2% (*n* = 4) White, 4.5% (*n* = 1) Asian, 18.2% (n = 4) multiple racial categories – one participant did not report) from three cities in a Midwestern, U.S. state participated in virtual, individual interviews. Most educators were college-educated; 36% (*n* = 8) held associate’s degrees (e.g., two-year college degrees), 41% had bachelor’s (*n* = 7) or master’s degrees (*n* = 2), typically in early childhood. The remaining 22% of educators either had completed some college without completing a degree (*n* = 3) or had completed high school or its equivalent (*n* = 2). Educators were 37.4 years old on average (*SD* = 8.8) and had been working in their current positions an average of 3.59 years (*SD* = 5.42).

Participants were recruited from a study of 106 educators (Brophy-Herb et al., 2023) focusing on educator stress and coping. All educators worked in infant and toddler classrooms in center-based programs in Early Head Start, Early Head Start childcare partnership programs, or for-profit childcare centers. About one-quarter (24%, *n* = 25) of the educators from the larger study responded to the invitation to participate in the qualitative interviews and consented to participate. There were no significant differences between educators who did not respond to the study invitation and those who did in demographic characteristics, such as age, *t*(94) = 1.32, *p* = .16, race (*p* > .05 for each *χ*^2^ racial group comparison, educational level (*p* > .05 for each degree status category), years in their current positions, *t*(96) = .37, *p* = .59, or in educator well-being, such as depressive symptoms, *t*(95) = 1.32, *p* = .16 or job stress, *t*(96) = −.24, *p* = .20.

Three educators who responded to the invitation were ineligible because they no longer worked in infant or toddler classrooms. All protocols and procedures were approved by the university research ethics review board (#17–1004), and the study was conducted in accordance with ethical standards as outlined in the 1964 Declaration of Helsinki. Informed consent was obtained prior to data collection.

### Procedures

Individual interviews were administered and audio-recorded on Zoom in the Spring and Summer of 2020. Three trained members of the research team, all identifying as white females, conducted the interviews. Two research team members, who were unaware of the study’s goals and did not conduct the interviews, transcribed all interviews verbatim for coding. Although the study was planned prior to the onset of the COVID-19 pandemic, data collection interviews began in 2020 as COVID-19-related closures were unfolding. To allow space for educators to acknowledge their experiences about the pandemic, we began the interview with questions about the specific impacts of the pandemic on educators’ stress and coping before moving on to the primary questions in the interview. In the main interview questions, educators were asked to think about their day-to-day experiences prior to the pandemic. The current study utilizes this set of questions (different from the pandemic-specific questions, the results of which have been published elsewhere; Green et al., 2025). Open-ended interview questions in the main part of the interview probed sources and characteristics of infant and toddler educators’ stress, the effects of stress and management of stress, and the experiences that help sustain educators in their work.

### Measures

#### Semi-Structured Interview Guide

Qualitative data were gathered using a semi-structured interview process. Educators responded to open-ended questions related to the relational stressors they experience, the impact of relational stress on their work, and how they mitigate the impact of this stress.

#### Demographic Survey

Information on educators’ age, race, education, work experience, and sex was gathered as part of the larger study.

### Data Coding and Analysis

As noted, educators’ responses to the COVID-related questions published in a separate manuscript (Green et al., 2025), along with findings from our prior implementation study (Stacks et al., 2024) illustrated the relationship-based nature of educators’ experiences and informed the a priori relationship-based framework with which we approached the coding in the current study. That is, based on prior experiences, we knew that relationships were important to educators and expected that the qualities of their relationships in the workplace would be present in their reflections of workplace stressors and experiences. We analyzed the data using thematic analysis (Braun & Clarke, 2006, 2021). As noted previously, our work is also grounded in the conceptual characterization of early childhood programs as complex ecological systems comprised of multiple relationships.

The first two authors coded the data, and the fourth author, who was familiar with the data from a prior study, reviewed the final themes. The first and fourth authors identify as white women of northern European ancestry. The second author identifies as a woman of Northern African descent.

We used the following six steps in the coding process, per Braun and Clark (Braun & Clarke, 2006, 2021): 1) become familiar with the data; 2) generate initial codes, characterized as a blend of conceptual terms, interpretation labels, and participant words/phrases that capture meaning; 3) search for themes, defined as generating or constructing initial themes and subthemes; 4) review potential themes, articulated as a recursive process in which themes are considered relative to their meaningfulness in capturing participant voices across the dataset; 5) define and name themes, during which themes are finalized; and 6) write the analytic results.

Coding was performed using a deductive, a priori relational framework and was carried out via Delve qualitative coding software (Ho & Limpaecher, 2024). As a first step, the first two authors independently read over five selected transcripts (step 1). They independently marked words and phrases in each transcript and kept ongoing notes about possible interpretations within and across transcripts (step 2) to develop initial codes (first-level codes). During a meeting to discuss the initial transcripts, the authors generated an initial codebook based on the first-level codes (step 3). For example, educators’ words and phrases such as “my purpose,” “it’s my passion,” “meant to do” evolved into the theme “Identity.” As another example, a code titled “other” was added to absorb any codes that did not match the initial codebook. During weekly meetings, the coders revised the codes in “other,” and if a new pattern emerged, a new code was added to the codebook (step 4).

Following this step, the coders continued coding the transcripts in a random order independently using the initial codebook and met weekly or bi-weekly over a period of five months to discuss coding and resolve any differences in coding via consensus. Both researchers entered qualitative notes throughout the coding process to ensure that participants’ voices guided the work (Padgett, 2017; Saldaña, 2021). The coders continued this process until all 22 transcripts (including the initial five) were coded and no new codes were detected, resulting in the final codebook (step 5). The primary coders then grouped the codes into themes and subthemes. Once the data were saturated and no new themes were evident, all transcripts were reviewed again to ensure themes accurately captured the major ideas expressed in educators’ interviews. Data saturation occurs when no new codes, themes, or patterns emerge (Corbin & Strauss, 2008). Finally, the coders created a written document describing the final themes and subthemes with quotes demonstrating each theme (step 6). As noted, themes and subthemes were reviewed by the fourth author. The results presented in the next section were derived from this analysis document.

### Findings

Thematic analyses of responses illustrated the ways in which educators find their work simultaneously stressful and rewarding (see Table 1 for the three themes and subthemes identified). Educators’ responses reflected both intrapersonal and interpersonal experiences that were largely embedded in their relationships with children, colleagues, and administrators. A key intrapersonal stressor was the cognitive and emotional toll of managing infants’ and toddlers’ varying developmental needs. Interpersonal stressors included balancing children’s many needs in classroom practices and interactions, holding multiple roles in their work with children and families (e.g., educator, social worker), and negative experiences in their relationships with administrators. Rewarding intrapersonal experiences, including their identity as infant and toddler educators, and rewarding interpersonal experiences, namely positive relationships with children and colleagues, were identified as themes. Findings from this study also suggest that educators intentionally reflected on the meaningfulness of their identities and positive, interpersonal moments with children and colleagues to buffer the impact of stressful experiences – including stressful relational experiences – on their classroom practices and relationships with children.

### Theme 1: Stressful Experiences

Educators’ stressful experiences were both intrapersonal and interpersonal. Intrapersonal stressors included cognitive and emotional demands on educators that were unique to working with infants and toddlers. Infants’ and toddlers’ high reliance on educators to meet all their needs, given their wide developmental ranges, meant that educators had to think and plan their curriculum carefully.

Interpersonal stressors reflected challenges in interactions, including implementing their carefully considered plans to meet infants’ and toddlers’ developmental needs while maintaining high-quality relationships with them. Moreover, educators described the challenge of balancing the multiple roles required in being an educator in an infant/toddler setting. Finally, educators described the ways in which interactions and relationships with their administrators were difficult.

#### Managing the Cognitive and Emotional Load in Working with Infants and Toddlers - Intrapersonal

Although educators enjoyed their work with infants and toddlers, they described mental drains, characterized by the cognitive and emotional labor of their work within the context of their relationships with children. Educators discussed the challenges of thinking about how to manage and meet infants’ and toddlers’ often widely varying developmental needs and interests, as described by these two educators:

“….like I said, you got eight kids with eight different developmental stages that they’re on… So it’s like, how can I actually be able to think for eight kids? To be able to give them exactly what they need, so that they can be progressing at the level that they need to be.”

“So, emotionally, as far as like that can take a toll on you. As far as just it’s trying to keep all of those, like, those times in your head, like, ‘oh this person used the restroom at this time or wait a minute, that person is going over there in the corner, that means he’s trying to have a vomit.’ So, your mind is just full of those particular times about the child and what he or she is about to do.”

Educators also described the mental and emotional labor associated with the immense felt responsibility for their work and their relationships with infants and toddlers. For example, one educator explained:

“I honestly, like, think we’re at like, we’re at the point where we’re shaping the kids. Like, ’cause we’re working with infants and toddlers and junior preschoolers and stuff, so they don’t, they do not know how to control their emotions. They don’t even know what emotions are or what they’re feeling, and we have to teach them that and help them figure that out. To me, that’s always been a big source of stress. Not that we have to do it, but am I screwing this kid up? That’s where, to me, like, the emotional and mental stress comes in.”

These excerpts from educators’ interviews indicate the short-and long-term nature of the mental work in infant and toddler caregiving. Educators thought about and developed plans to support infants’ and toddlers’ needs and development on a daily basis. Educators were also keenly aware of their potential impacts on children’s well-being and development and felt a strong sense of responsibility for children’s healthy growth.

#### Challenges in Interactions – Interpersonal

Educators described three types of interactions that were stressful to them. First, as noted previously, educators think deeply about planning how to meet infants’ and toddlers’ needs. In turn, the implementation of those plans is stressful. Second, educators also explained their perceptions that they held multiple roles in the lives of children and families (e.g., educator, social worker, family advocate, etc.). Finally, educators identified interactions with administrators, often “upper” administrators to whom they felt under-valued, as stressful.

##### The Demands of Balancing Infants’ and Toddlers’ Needs

Educators articulated many examples of the ways in which they perceived providing infant and toddler care and education as more demanding than working with older children. Many of these differences were focused on infants’ and toddlers’ dependency on their educators, particularly during routines like mealtimes when multiple child needs required educators’ attention at the same time. Educators also noted the challenges in understanding infants’ and toddlers’ needs communicated nonverbally. The following two educators articulated their experiences:

“Because for younger children, especially in my area, I deal with the toddlers and that’s from one and a half to three and you’re dealing with changing a pull up, you know, potty training, and constant movement. So, you rarely have a chance to sit down. You’re always on the move because in my particular classroom, it’s me, and my teaching team partner, with eight children.”

“I think, honestly, I think I’m gonna say it’s when they cry. That’s the hardest part because you don’t know, oftentimes, you don’t know, why they’re crying. If they’re sad or if they’re really tired or if they’re really hungry and you know with infants that’s all - you know the little ones that’s a lot of what they do is cry and you’re trying to figure out what they’re thinking, and what they need, anticipating their needs….”

Educators described the challenges encountered in managing mealtime experiences. For example, one educator shared the following example:

“For myself, it’s stressful because we serve meals family style. So that means that in the very beginning, we have to teach them how to pass the bowl of food or how to use the spoon to get a serving and with children they don’t have the concept of quantity. So just basic things like pouring milk - so If you pour the milk from a small carton into a cup, they’re just gonna overflow the cup. So, you have to really teach individually each child how to do certain things.”

These excerpts suggest that educators may experience more stress in managing infants’ and toddlers’ needs during caregiving routines than in supporting children during play. Educators’ quotes also point to the in-the-moment responses in which educators must “figure out” and enact support for infants and toddlers during routines.

While most participants felt connected to the children in their classrooms despite the demands of meeting multiple needs, others felt stress and worry when they had a difficult time building relationships with children. For example, one educator explained:

“For me I think my biggest struggle is when I’m not connecting as well with the child. I’m thinking back to just some children I had struggled with a little bit that were a little bit more emotionally charged individuals in general. And that is just hard as you wanna help them, but again every interaction is frustrating and just finding that balance in your head is a little bit, I would say, more mentally exhausting than yeah just overall.”

This quote presents another lens on meeting varying needs in interactions with infants and toddlers as the educator references the child’s temperament. Note, this educator’s comment also highlights the link between the intrapersonal stress of mentally managing children’s needs and the interpersonal stress experienced at times during interactions.

##### Managing Multiple Roles in Relationships with Infants, Toddlers, and Families

Educators described perceptions of their roles as including not only educational and caregiving practices but also responsibilities related to children’s health and family well-being. Educators often felt that others do not understand the complexity of their roles. One teacher articulated, “*I don’t think that they understand exactly how many hats we do wear*.” Another educator also used the hats metaphor:

“Well, for me, I feel like for me, it’s hard because of all the things you have to do as a teacher. Like, I have to explain to everybody I am not just a teacher. I’m a teacher; I’m a mother; I’m a nurse, and I’m like everything born in one. It’s a lot. As a teacher, you want to be a teacher, and like I said, wear many hats, but you also have to be careful with them hats because you don’t want to, you know, you don’t want to go beyond your boundaries with the parent or whatever.”

Educators in this study also spoke about their worries for families, and while they valued their role as resources for parents, this was also a source of stress, as described by two educators:

“I always try to keep an open door too with my parents and let them know that I’m here to talk with them, and that goes back to the original questions you were asking ’cause I take on a lot of their stresses. And when I say I have an open door and they can come at me anytime, they do. That causes extra stress, but I feel like at the same time, that’s my position to try to help.”

As a teacher for younger kids because they’re younger. They’re more, you know, I think they’re more… there’s just more interactions between you and the parents and I think that can be a little bit stressful too.

Infant and toddler educators also worried about how children might be impacted by what families were coping with at home, and they were concerned about building positive and supportive relationships with families. Two educators explained:

“I think seeing a child go through things at home. They can’t explain to us what’s going on. And the way we handle it is—see, we’re not supposed to ask the parent, but most parents kind of tell us what’s going on at home, and we just be there for that child or that parent.”

“Um because some days, you know, the parent will just throw the child in screaming and hollering, and the parent look like they’re stressed out. So if you have a good communication with your parents and they’ll open up about what’s going on. ‘Well, little (CHILD) didn’t eat this morning or he didn’t get enough sleep…” Then I can better assist that child.

Educators’ commitment to children is evident across their comments as they described the responsibility they felt to “wear many hats” as they serve both the children, meeting their multiple needs (physical care, support for learning and development, emotional needs), and the families (e.g., as educators, as family advocates). As seen across themes, educators’ comments here also speak to the relationship-based nature of their work, for example, in the quotes above, they describe the importance of high-quality educator-parent communication in their efforts to best support infants and toddlers.

##### Quality of Relationships with Administrators

While most educators in this study described their interactions with their direct supervisors positively (e.g., “*she pretty much helps me get through it…*;” “*I can always go to her*;” and *“I like what I do. Actually, I love what I do. And I think the supportiveness of my boss is helpful”)*, others described the stress experienced when feeling disconnected from the upper levels of administration. Educators described feeling unseen and unheard by administrators, shared perceptions that administrators did not fully understand or support their everyday work, and they identified poor communication between administrators and educators as an additional stressor.

##### Feeling Unseen and Unheard

Many educators suggested that their perspectives as educators were not consistently considered in larger administrative decisions or in their interactions with administrators. They expressed frustration when administrators don’t take the time to listen to their needs, such as the stress described by the two educators below:

“It gets a little exhausting when you have these different people telling you, ‘Try this, try that.’ And you are taking the suggestions, but they’re not really listening to you.”

“Woo, I don’t know, I just don’t think [administrators] take the teachers into consideration when they expect us to do a lot of things which takes, to me, takes away from the care of the kids.”

Educators’ quotes speak to the multi-level, systemic organization of large early childhood education programs, such as Early Head Start and its affiliated programs. This idea also extends to the perceived lack of understanding, which is addressed next.

##### Lack of Understanding and Support

Many educators articulated a disconnect between their daily experiences in the classroom and administrators’ understanding of educators’ classroom experiences.

“I wish that they knew, understood the other piece. Like the personal side to like the extra stuff that I have to, that I do to accommodate my families. That part. Like all they look at, you supposed to do this, you supposed to do that, and that’s it. But it’s bigger than that, it’s just not, it’s just not that.”

“Ok, so I feel like they should be more in the classroom. I feel like they should be more in the classroom, so that way, they understand what we’re talking about. That way, they understand how things are doing.”

Similarly, educators described a lack of support when their administrators were not looking out for their needs. Sometimes, support could have come in the form of concrete support, as suggested by the educator below, who was responding to numerous distressed children in her classroom.

“You know, if you’re outside in the hallway and you hear [children] crying, could you at least check to make sure they’re okay? Or you know, ‘Do you need help?’ Sometimes, you know, I saw four people pass by and nobody checked on me. So that kind of a thing.

Another teacher described a lack of support when she was caught off guard by her administrator’s unexpected behavior:

Knowing that [the directors and administrators] have your back. And they’ll stick up for you, and they’ll do all that stuff. That actually is a huge thing ’cause we’ve had a couple instances where we got together as, you know, myself and my boss and we talked about things that we were in agreement on the way this is going to be handled and then she totally turned around and does something completely different and said something completely different to the parents, and then you’re hanging in the wind and you’re like, ‘wait a minute, what just happened?’”.

These examples show that educators felt that administrators did not fully understand or value what everyday classroom life is like, and that resulted in a perceived lack of support. Educators believed that knowledge of their daily experiences was an important context for the practices and policies educators were asked to implement, as well as key contexts for understanding the complexity of infant and toddler care and education and the toll it can take on educators.

#### Poor Communication

Educators described the lack of effective communication. One educator commented, “…*it’s like we’re speaking two different languages sometimes*.” Other educators explained:

“I would say I find the most stressful thing is communication. The lack of effective communication. Instead of it being kind of everybody being on the same page, you will possibly hear one thing and then another thing before actually getting a clear picture of things.”

“And sometimes there’s not a lot of communication between the people up there and the people actually working with the children.”

Educators also expressed their desire for open communication between administrators and educators. One infant and toddler educator shared:

“I would say just open communication. I like to see my bosses in my classroom. I like them to see me in action so that I can get direct feedback on what I’m doing and so they know the work that we do.”

Like other themes, the comments above show examples of the relationship-based nature of infant and toddler educators’ experiences. Clear communication is an important element of a healthy workplace, interpersonal experiences, and relationships. Notably, poor communication quality may contribute to educators’ sense that administrators do not fully understand their work and experiences.

### Theme 2: Rewarding Experiences

In addition to reporting on stressful relationship-based experiences, educators reported experiencing a great deal of both intrapersonal and interpersonal rewards. First, educators described their work with children as a calling that is personally meaningful and rewarding to them. They valued the impacts they have on children’s development and viewed their work and their influence on children as a core part of their identity. Second, educators identified positive interactions with children and colleagues as rewarding relational experiences in their work.

#### Identity – Intrapersonal

Educators described their work with infants, toddlers, and families as a calling that gives their lives purpose - the work is part of who they are. This purpose was central to remaining in their careers. These two educators help us understand how critical this internal sense of commitment and passion for their work is to them:

If that’s your purpose to work with children in a hands-on, engaging, way; to help them grow and develop, to nurture children– if that’s your purpose, your calling, your passion, then, then, then you’ll do it. If it’s not, you won’t. You won’t because it will be like, ‘No, this is too much’.” -

“What helped me stay in the field is the children because I do this for them. You know what I’m saying? This is my passion. I always wanted to be an educator. I always wanted to educate children.

Moreover, educators in this study valued their roles in supporting infants’ and toddlers’ early development, and they viewed their work as contributing to a healthier society. These three educators explain that the reward for their work is not financial; it is the difference they create in the lives of children:

“Like we care about the kids. We’re not just here for a paycheck. Because if we were here for just a paycheck, we wouldn’t be here. It’s not worth it. And so, we absolutely love what we do. And I think a lot of people don’t really understand that. Like this is a labor of love, you know.” “We’re not doing this because we’re getting paid the big bucks because I’m not, you know. I’m not doing it for anything else. I’m doing it because I love kids, and this is what makes me happy and I get to help shape and mold little kids to be, you know, the best part of society.”

“I just think that there’s so many wonderful teachers, too, out here. And it’s not about the money at all. It’s definitely not about the money. It’s basically about making a difference in a life.”

Educators in this study help us to understand that their identity as professionals who positively impact young children’s development (thereby contributing to a healthier society), is a core intrapersonal experience that needs to be recognized and respected. Acknowledging this commitment to the field is essential to sustaining an early childhood workforce that is invested and motivated to deliver high-quality practices and shape children’s development in positive ways.

#### Interactions – Interpersonal

In this study, educators described interactions with children and with colleagues as important to their work. Enjoyable moments in relationships with children and supportive relationships with colleagues were especially rewarding and promoted positive feelings about their work.

##### Enjoying Moments with Children

Educators’ enjoyment of children was articulated by their positive feelings about their work and cherishing connections with infants and toddlers, both in the present and over time. For example, these two educators’ sense of fun and pleasure in working with infants and toddlers is clear:

“I think it’s just the nature of being a teacher. If you love being with kids and you love what you’re doing with them, being with them on a daily basis is natural for you. It’s something that you like doing, you know. It’s something that you wanna do. You wanna be with them. You wanna come and so you kind of focus on them.”

“You know, it’s what I wanna do. I love littles. I love seeing you know, they are so cool. They don’t have a filter and they’re not to that point yet, so I love all the crazy randomness that comes along with this job and seeing through their eyes and helping them learn how to problem solve and stuff like that. Like helping them learn their skills. That to me is what is keeping me here.”

Positive feelings also extended to their efforts to build relationships with children. One educator shared how enjoyable moments can develop even when working with a child with challenging behaviors:

“Thinking about moments with children - I had a little girl that, she was, her, she was terrible everyday. Throwing tantrums, chairs. And I was trying to figure out how to reach her. And I combed her hair one day! And after that, she’s been fine. But even now she got a teacher and her teacher reached out to me. She said, you know when her hair’s not done, she has a very bad day. So, I have actually combed her hair. Like on different occasions to make sure that because she was having a really bad day. And it might be something as simple as me just taking the rubber band out and doing like this and putting it back in and then telling her she looks beautiful.”

Educators also spoke about their long-term joy in interacting with children over time and shared their hopes that children remembered and cherished their experiences with them. For example, in describing her interactions with infants and toddlers, one educator shared:

“Um the rewarding side of it, you know, that you are molding these young children to be successful, and you want them to look back on you, you know, down the line and say, ‘That was my teacher. That was an awesome teacher when I had that teacher um when I was younger.’ You know, just going back to say that you did make a difference. It’s rewarding trying to, you know, be kind and be um uh, you know, just being there for the families and children, so.”

Findings also suggest an interconnection between educators’ intrapersonal commitment to children and the interpersonal rewards experienced in their relationships with infants and toddlers. This was illustrated by an educator who expressed their love for their work and how it makes them feel (intrapersonal) and how this positive feeling was associated with positive and successful interactions (interpersonal) with children in their classroom:

“I just, I just think that I’ve been doing it for so long that I love it. And I, I like the reactions of knowing that I have an impact on what this child is going to know in the future. Like I did, I helped you with that. I taught you certain things about your development, your physical, your social emotional. I taught you things that you might not have taught you, but just being in my classroom, and playing in the house area, helped you learn certain things. I also helped you learn how to communicate and how to, you know, form sentences and words and expressing your feelings. Like to know that I had that impact on somebody is what keeps me teaching.”

Collectively, these quotes speak to the many ways educators build relationships with infants and toddlers and enjoy interactions with them in the short- and long-term. Quotes also showed educators’ desire to see the world through children’s eyes, to use caregiving routines, such as hair combing, to exchange positive moments of connection with children, and highlighted their hopes that their interactions have long-lasting impacts.

#### Enjoying and Benefitting from Relationships with Colleagues

Most educators described positive, supportive relationships with colleagues, particularly their co-educators. As these educators explained, high-quality relationships were a benefit to them, and their colleagues served as important sources of positivity and support, and were sounding boards for ideas to benefit children:

“So, I think that sometimes we try to rely on each other to try to, kind of, relieve this stress. My teacher partner and I just come up with different ways, so that we don’t go home both tired and stressed. So, we kind of piggyback on each other and if we feel like we need each other, need help, we just ask each other basically, you know, what do you think we can do or how can we make it better or can you help me do something?”

“Um, and a lot of times also it’s, uh, connecting with other teachers because sometimes you think it’s only you that, you know, that’s dealing with it. But when we come together, you know, we out like, ‘Wow. You dealing with this too? How do you deal with it?’ You know, collaborating with co-teachers and you know, finding out that you know, you not the only one that’s feeling like this. But, so how can we fix this?”

Participants in this study kept infants and toddlers at the center of their work when considering relationships with colleagues. This educator discussed the importance of knowing that trusted colleagues were close by and would help with the children if needed:

“I think just knowing that I’m not in it alone. We have three staff in the room right next to us. In our room I have two other people who are working with me, so knowing that we’re not in it alone and you know what I mean. You can always count on the other person saying they can you go do this, and, you know, they can you go pick up the child from the crib, you knowing that they can do it. There’s always somebody in the room with you to kind of to back you up.

In this subtheme, educators’ comments underscore the importance of positive relationships between educators. These relationships contributed to educators’ well-being and to educators’ practices. They rely on colleagues for support to provide high-quality experiences for children.

### Theme 3: Use of Intrapersonal and Interpersonal Experiences to Buffer Stress

Educators intentionally recalled and reflected on positive interpersonal experiences, primarily cherished moments with children, to manage stress. For example, one educator described how she would “*absorb myself into the kids*” and their interactions together to let go of worries. Other educators identified how focusing on the fun, enjoyable moments with children helps them cope with stress:

“You focus on the fun parts trying to get them to smile, trying to, you know, get a little laugh out of them and I think that’s a lot to help with teachers stress because, you know, I mean those little interactions I think can help get you through the day.”

“And sometimes if I am super stressed, just throwing it out to the side and sitting down interacting with the kids brings me joy, calms me down and gets rid of some of my stress. So that is how I do that.”

“I guess just finding moments to be happy every day. I think that’s really important to leave work with memories and to make sure your kids left with happy memories every day. It makes it easier to come back every day.”

Holding on to positive experiences with colleagues was also a helpful strategy for buffering stress, as evidenced by this educator:

“Try to laugh a lot with my coworkers. My immediate coworkers, we try to see the fun in the situation and if we see a kid doing something fun, you know point it out to the others, seeing what the other teachers are doing and you know just having fun with the kids and laughing with them and yeah I think that’s a great way to relieve our stress.”

Excerpts in this section show educators’ intentional use of positive intrapersonal (identities as infant and toddler educators) and interpersonal relational experiences (moments with children and colleagues) to sustain them in their high-quality practices with children and protect their relationships with children against the stresses that could infringe on their interactions. Educators’ quotes also show how these pleasurable reflections are related to job retention – “*It makes it easier to come back every day*.” However, it is also important to note that these intentional efforts reflect yet another type of stress for educators. As educators suggested in the earlier quotes in this section, “…*you can’t, you know, express your frustration or anger to the kids. You still have to put that on the back burner*.”

To that end, educators described their intentional efforts to prevent stress from negatively impacting their relationships with children and their classroom practices. This finding suggests that educators are intentional in their use of what they view as intrapersonal (i.e., this work is a calling and gives them purpose) and interpersonal relational experiences (i.e., moments with children and colleagues) to buffer the effects of stressful experiences in their work.

The following quotes from educators highlight the ways in which their core identities as infant and toddler educators, that is, the intrapersonal rewards of the work, help them manage stressful experiences. For instance, in describing the management of stress, two educators told us:

“…then having those breakthrough days, as you would say, or those great moments with kids. I think you recognize when they happen and know that you’re in the right career.”

“Coping with the stress, like I said, just always going back to remembering why I’m there and know that it’s for the families, because we have families that are less fortunate, like poverty, and they’re families in inner cities, so we deal with a lot of families that are going through a lot of things.”

This recognition of their sense of calling was also paired with an intentional centering of children in their work and educators’ intentional efforts to prevent stress from impacting their interactions and practices with children. For instance, one educator explained:

“I think we just realized that the other day we’re here for a reason. So, we just learn to just bag up our issues or bag up our emotions and just kinda set it aside in that moment so that we can get through what we need to get through and teach our children what we need to teach them and have their support while they are in our care. Because at the end of the day, it’s all about making sure that child is safe and that were being able to provide as much as we can while they’re in our care. That’s really all that matters.”

The quote above demonstrates educators’ self-regulation strategies, used in the service of maintaining high-quality, responsive practices. Other educators elaborated on their intentionality about preventing stress from impacting the children and their practices with children, as explained by the two educators below:

“Are you going to let it affect your performance in a poor way which, in return, will affect the children? And you make a decision so… and I think for the most part, most teachers make the decision that they would not neglect what they give to the children.”

“So, I think we have just programmed ourselves to just be. Because this is a child! A lot of things they can’t control so it’s like, even if I’m stressed about it, I can still perform. You know it takes a lot for me personally to. I can be under a lot of stress, but I’m still going to be able to be at work and I’m going to be to do what I need to do.”

In this section, we have demonstrated educators’ intentional use of positive, and often relational, intrapersonal and interpersonal experiences to manage stress and to mitigate the effects of stress on children and on their responsive practices with children. Educators’ comments also suggest the complexity and multifaceted nature of their relationships in the workplace.

### Synthesis of Educators’ Relationship-Based Work with Children and Families

**Fig. 1:**
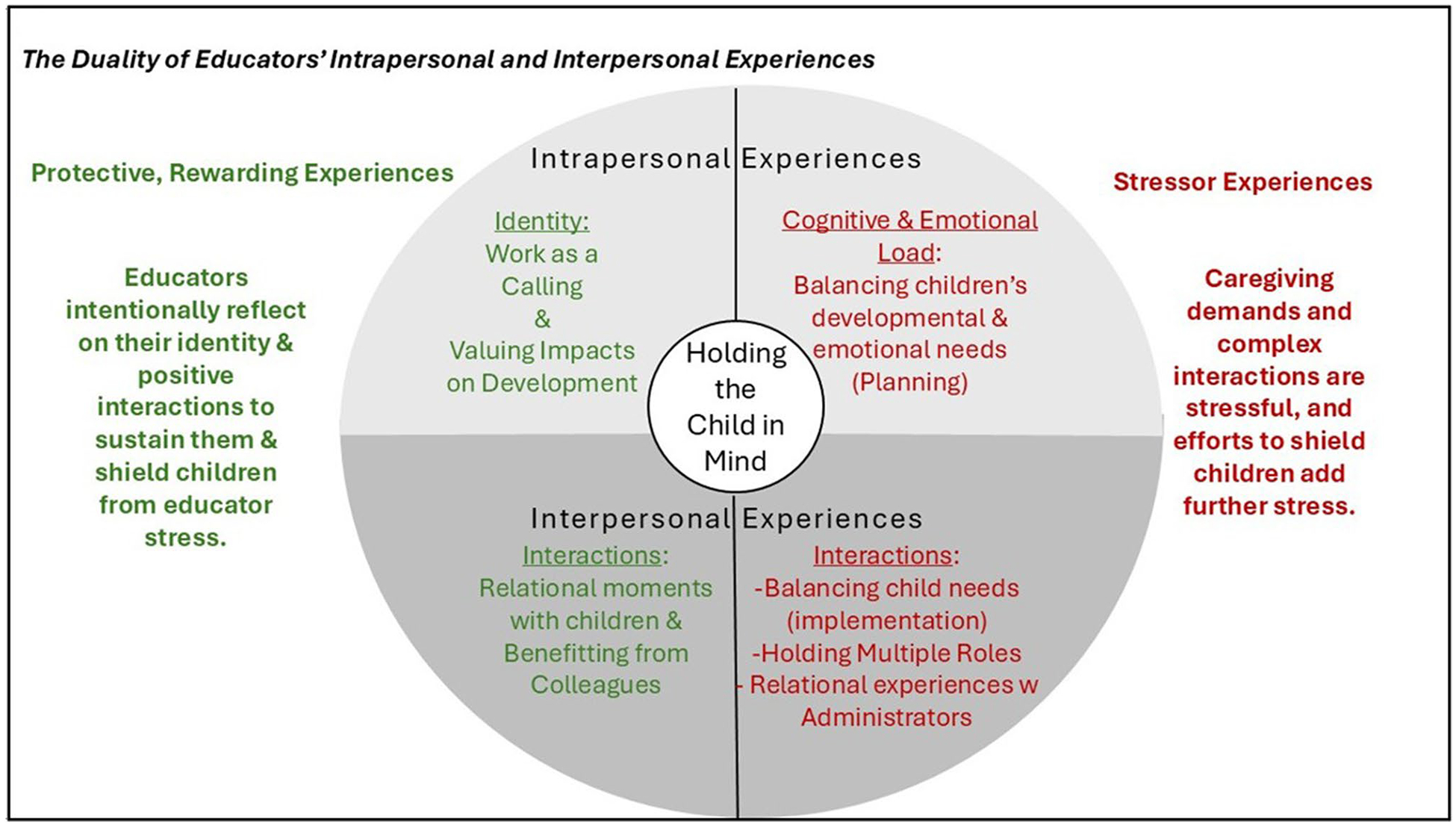
The Duality of Educators’ Intrapersonal and Interpersonal Experiences. The figure summarizes infant and toddler educators’ descriptions of their rewarding and stressful intrapersonal and interpersonal experiences at work, including educators’ intentional use of positive intrapersonal and interpersonal experiences to buffer stressful experiences. The figure also reflects educators’ centering of children in their work and work experiences. Educators acted intentionally to shield children from stressors

Reflecting on educators’ comments about their experiences led us to create a model that illustrates how they described the associations between intrapersonal and interpersonal rewarding experiences and stressful experiences (see Fig. 1). The themes suggest that educators continually hold children in mind at the center of their work; that is, the “why” and “how” of their work is centered around supporting infants and toddlers. Educators’ positive experiences were largely characterized by their commitment to and enjoyment of children, while challenging experiences often reflected stress experienced in trying to provide the most optimal experiences possible for infants and toddlers. Educators were clear in their efforts to use positive intrapersonal and interpersonal experiences to buffer children from any effects of their stressors; yet, these intentional efforts created another form of stress in the relationship-based work of ECE.

## Discussion

Findings underscore the duality of infant and toddler educators’ work with children as simultaneously stressful and rewarding. Educators identified intrapersonal demands such as cognitive and emotional load alongside rewarding intrapersonal characteristics, such as professional identity. Interpersonally, they reported stressors related to meeting children’s needs, balancing multiple roles with children and families, and interacting with administrators while also emphasizing the rewards of relationships with children and co-educators.

While some results, such as the duality of infant/toddler caregiving as satisfying but stressful, are consistent with the existing literature (e.g., Hamel et al., 2025), our findings suggest that well-being may be better understood when keeping the co-existence of both positive and stressful feelings in mind. Moreover, we focus on the following three novel, key findings: 1) the framing of educators’ work experiences from intersecting intrapersonal and interpersonal perspectives; 2) educators’ explanations of the nuanced, challenging interactions with administrators, primarily “upper” administrators with whom they had less frequent contact than their daily supervisors; and 3) educators’ intentional use of positive intrapersonal and interpersonal relational experiences to cope with their stress, and to shield their classroom practices and the children from the effects of their stress. We discuss these findings in the context of emerging work from Jeon et al. (2026) on relational well-being and informed by the understanding of ECE programs as complex, ecological systems comprised of multiple relationships.

### Intersecting Intrapersonal and Interpersonal Experiences

#### Stressors in Educators’ Experiences

Educators spoke about the heavy cognitive and emotional labor involved in planning, implementing, and balancing individualized developmental supports for infants and toddlers (intrapersonal experiences) during interactions with children (interpersonal experiences). This finding aligns with current literature that early childhood educators must adapt to children’s unique and rapidly changing development across learning domains (McGoey et al., 2022). These demands are compounded by the need to support many infants or toddlers at once, each of whom is heavily dependent upon their caregivers to meet their needs. Interestingly, educators described stress related to responding in the moment. They had to “figure out” and respond sensitively to children’s behaviors that did not have a clear cause or obvious solution. Educators also described “group” experiences, such as mealtimes and rest times, as more challenging than flexible play, perhaps because multiple, urgent needs surfaced simultaneously. These situations reflect the cognitive load and emotional labor of balancing the unique needs of multiple children while sustaining sensitive, responsive interpersonal interactions that promote healthy development.

Managing the cognitive and emotional complexity of early childcare and education requires sophisticated skills (Malhotra, 2022), particularly given that educators work so intentionally to prevent stress from impacting their relationships and practices with children. The challenges of doing this work have perhaps become even more significant as the landscape of ECE has evolved to provide more optimal learning environments for a range of learners, for example welcoming and supporting children with additional support needs, children who have experienced trauma, children from wide-ranging cultural backgrounds, any of which require high levels of awareness, knowledge, and skills. Yet, most educator preparation programs do not include enough content on working with infants and toddlers (Gilken et al., 2023). The lack of attention to infant and toddler educator preparation and professionalization may also reflect the continued problem of feminization of early childhood work, including the devaluing of early childcare and education (Fairchild & Mikuska, 2021), and the problematic characterization of early childhood practices as “natural” or “innate” (for females) requiring little skill or training (Malhotra, 2022). On the contrary, focus on the relational nature of early childhood educators’ experiences, and the intentionality with which educators embrace relational moments, points to their skill and sophistication in their work.

Another interpersonal source of educators’ stress is the role they play in responding to families’ needs, which often fall outside of their area of expertise. Interestingly, while educators clearly valued families, they did not identify their relationships with families as highly rewarding. We wondered if perhaps this finding might reflect a form of depleted cognitive and emotional resources. That is, educators intentionally countered the cognitive and emotional demands of supporting infants and toddlers with the joy and rewards of working with them; and this balance requires extensive energy. It is possible that educators simply have less energy available to invest in balancing the demands and potential rewards in their more distal relationships with families. Further, some areas of support, such as connecting families with resources, may fall outside of educators’ roles and responsibilities. In these areas, programs likely hold more of the responsibilities to have information on resources, for example, available for families.

Relationships with colleagues were overwhelmingly experienced as positive, while the opposite was true for relationships between educators and administrators. The most robust interpersonal stressor was educators’ challenging relationships with “upper-level” administrators with whom they worked less directly. These educator-administrator dynamics are consistent with both relational well-being and workplace culture/climate in the ecological model of holistic early childhood workforce well-being (Jeon et al., 2026). The current study extends the existing literature by identifying relational disruptions that weighed heavily on educators. In particular, educators were troubled by feeling undervalued, unsupported, and misunderstood, paired with concerns about communication clarity and consistency. The first three characteristics of problematic relationships underscore the necessity of cultivating belongingness, a critical aspect of diversity, equity, and inclusion practice in organizations (Mor Barak, 2015) and an indicator of holistic well-being among educators (Jeon et al., 2026). Our findings are consistent with those of Kim (2024) who reported that to feel supported, educators needed to feel that they were valued, appreciated, and respected by administrators. This sense of educator belonging, central to workplace culture, is understudied among educators given that existing work tends to focus on children’s sense of belonging (Erwin et al., 2024; Johansson & Puroila, 2021; Singer & de Haan, 2010). In a relationship-based setting like ECE, attention to all important relationships is critical to well-being.

Findings regarding problematic lack of communication with administrators were not surprising as other recent work also reported that poor communication negatively impacts educators’ work (Vesely et al., 2024). A more novel contribution of our study is educators’ perceptions that administrators did not fully understand their day-to-day classroom experiences. Participants in this study expressed a desire for administrators to spend more time in classrooms, noting that it could enhance feedback communication and could also deepen administrators’ understanding of the classroom environment. We suggest that administrators’ understanding of educators’ day-to-day experiences— and likely a shared understanding of each other’s roles between administrators and educators—characterizes an essential intrapersonal component not only of the workplace climate but also of relational well-being.

This study reinforces the need to support and strengthen administrator-educator relationships, particularly those between upper management and educators. We often expect educators to provide children with nurturing, responsive relationships that foster a sense of value and belonging—yet we don’t always expect the same care to be provided to educators themselves. While we invest heavily in professional development for both educators and administrators, it would be valuable to determine whether and how much training is focused on cultivating the relationships between them to promote a strong sense of belonging among the whole organization.

#### Rewards in Educators’ Experiences

While some intrapersonal and interpersonal experiences were characterized as stressful, educators also described positive, rewarding intrapersonal and interpersonal experiences with children and colleagues. The importance of educators’ identities as professionals immersed in relationship-based work was articulated clearly by educators. Very few studies of educator well-being have examined professional identity as an indicator of well-being, although identity reflects a conceptually hypothesized component of professional well-being (Jeon et al., 2026). Importantly, our study suggests that educators’ identity, an intrapersonal experience in which they see their work as a calling to positively shape children’s development, interacts with their interpersonal experiences in shared moments with children and colleagues, elements of relational well-being. Educators’ identities fueled their enjoyment of interpersonal and relationally rewarding experiences with children and colleagues. In turn, these positive relationships reinforced their identities as infant and toddler educators. The bidirectional paths between intrapersonal and interpersonal experiences as described by educators align with the proposed, but understudied, dimension of relational well-being described by Jeon et al. (2026). These researchers describe relationships with children, families, and coworkers, interpersonal skills, personal relationships, and communication skills as relational well-being, and place it as central to holistic well-being, which is the center of their model. Our findings contribute to the work on educator well-being, and particularly the new work on relational well-being, by defining intrapersonal and interpersonal dimensions of well-being.

The positive psychology literature, including the literature on psychological rewards, provides another relevant lens through which to view educators’ rewarding intrapersonal and interpersonal experiences. Psychological rewards are the intangible benefits individuals (often in human service professionals) experience, such as personal satisfaction and enjoyment (De Gieter et al., 2008; Lee, 2019). Although the term “psychological rewards” has been rarely applied in the early childhood education literature, the concepts are represented in recent literature. For example, joy and positive feelings about work are linked with buffering and easing educators’ stress and exhaustion (Chen, 2025; Kwon et al., 2020; Little & Karaolis, 2024). Moreover, Avola and colleagues (2025) applied a positive psychology model in a recent scoping review to organize and summarize various educator well-being intervention activities for early childhood educators through higher education instructors. Their scoping review showed that most educator well-being inventions are focused on individuals rather than on relational experiences, highlighting the need to include relationship development in support efforts (and in intervention evaluation efforts).

Moreover, the concept of psychological rewards also aligns within Jeon and colleagues’ model of educator well-being (2026). Specifically, psychological rewards are reflected across elements of educators’ psychological well-being (e.g., intrapersonal experiences of personal satisfaction and psychological capital), professional well-being (intrapersonal experiences of joy and happiness in their work with children and colleagues), and relational well-being (interpersonal experiences of high-quality relationships with children, families, colleagues, and supervisors). Borrowing from the psychological rewards literature offers another useful conceptual tool in characterizing the complexities of educators’ intrapersonal and interpersonal experiences, although the field will benefit from additional conceptual work in integrating current models of well-being with emerging concepts related to intrapersonal and interpersonal psychological rewards and experiences.

Our findings on the duality of educators’ positive and challenging intrapersonal and interpersonal experiences with children offer insights into how educator well-being, including relational well-being, may be further conceptualized and studied. Educators have taught us that both stressful and rewarding experiences—as well as the feelings that come with them—co-exist everyday and are not easily separated from one another. Studying relational well-being and other forms of educator well-being authentically is difficult in the absence of simultaneously addressing the inverse of educators’ positive experiences. Future models of educators’ stress and well-being should examine this duality of their experiences and the associated feelings. Perhaps more importantly, a key indicator of well-being may be the extent to which the coexistence of the work’s stressors and rewards is balanced. In continuing to build and refine models of well-being, professional development programs may be better informed to enhance the positive, rewarding experiences, while offering supports relative to educators’ more challenging experiences.

#### Use of Intrapersonal and Interpersonal Experiences to Buffer Stress

Educators’ intentional use of both intrapersonal and interpersonal rewarding experiences to cope with stress is a novel contribution to the educator stress and coping literature. While other studies have linked commitment and sense of purpose with lower turnover intentions (e.g., Vicente et al., 2025), our study extends prior work by identifying a potential mechanism underlying these associations. Specifically, our findings suggest that educators intentionally think about their purpose during stressful moments as a coping strategy, which may help explain why sense of purpose is associated with continued commitment in their profession despite ongoing stressors. Moreover, despite the stressors related to careers as infant and toddler educators, educators’ commitment to their work with the youngest children may have protective benefits for them. For example, Bryant and colleagues (2023) found that working with infants and toddlers specifically, as compared to working with older children predicted educators’ retention in the workforce (along with positive work climates). Integrating Bryant’s work with our findings raises the intriguing possibility that there is something uniquely rewarding about the types of connections educators form with young children. Deriving a better understanding of these connections can inform efforts to sustain educators despite the stressors inherent in the work.

Our findings on the importance of positive connections with children and colleagues align with the results from a recent scoping review of well-being among Head Start educators (Wilson et al., 2023). In this review, researchers reported that a sense of community and connectedness, positive feelings about their workplaces, trust in workplace climates, and positive relationships with colleagues, supervisors, and children were related to educators’ higher well-being and lower turnover. Our study contributes to this literature because educators’ active, intentional use of positive, relational moments with children and colleagues as a specific, intentional coping strategy may help to explain one mechanism through which positive feelings and connectedness are related to well-being. As another example, a recent study on early childhood educator joy found that educators “thoughtfully and intentionally looked to the child as the source of joy or the recipient of joyful learning” (Little & Karaolis, 2024; p. 86). However, this study was focused on the concept of educators’ experiences of joy rather than the employment of joy as an active coping strategy. Our study findings, however, help to explain how educators’ intrapersonal and interpersonal positive experiences are related to their well-being.

The fact that educators naturally gravitated to using these rewards as a way of coping with the stressors suggests that they may be ideal targets for professional supports that have thus far been overlooked. There are several possible strategies to boost these intrapersonal rewards, including interventions informed by positive psychology and reflective supervision consultation models. At least within Early Head Start, infrastructural supports, such as coaching, are in place and may benefit from additional content on positive relationships. Fostering peer mentoring and collaborations between educators within ECE programs and perhaps even across ECE programs might be additional strategies to consider, given educators’ reliance on positive relationships with colleagues. Similarly, boosting interpersonal rewards could be informed by positive psychology frameworks found outside of the ECE but applicable to work with educators. For example, relational savoring (Borelli, 2024) is a strengths-based strategy grounded in positive psychology (Fredrickson, 2001) and attachment theory (Bowlby, 1973) that involves helping individuals remember and re-experience positive moments of interpersonal connection. Relational savoring has been used as a strategy to improve well-being and relationship quality within parents (Borelli, 2024; Borelli et al., 2022; Burkhart et al., 2015) and is conceptually relevant to the relational work occurring in ECE contexts. Helping educators savor moments when they do feel supported by their supervisors may help them recognize positive aspects of educator-administrator relationships that they may otherwise miss. Similarly, helping administrators, particularly upper administrators, connect with educators and relish those moments may help improve and sustain their relationships.

### Strengths and Limitations

Strengths of the current study include the racially diverse sample of educators involved in this study which contributes to greater generalizability. The fact that the themes reported reflect commonalities across participants enhances confidence in the findings. We acknowledge that the interview questions employed in the current study were largely designed by white researchers. Although the open-ended questions were driven by the researchers’ informal conversations with educators in the larger study, an approach involving community-engaged development of interview questions would enhance confidence that the resultant product addresses the most relevant topics.

Future studies may consider exploring additional questions on the variation in relational rewards and stressors among early childhood educators and how these affect stress levels, burnout, and career intentions. The small size of the current study did not allow us to analyze results separately by educator characteristics, such as age, race, or length of time in the field. For example, among educators of older children, racialized school climates and micro-aggressions are related to greater burnout (Mahatmya et al., 2022). Further, data show that early childhood educators who are Black and Hispanic/Latino have lower average hourly wages on average compared to their white peers (Liu et al., 2025), which could certainly contribute to stress, burnout, and turnover. These intersections should be thoughtfully considered in the study of belonging within educational organizations, and in interventions to promote belonging among those in relational fields of work.

Contextual characteristics of the study design, namely the timing of the study and the structures of the programs in which educators worked, may limit generalizability. Specifically, data were collected in Spring 2020, just as the COVID-19 pandemic-related shutdowns were occurring. The experiences of this time could have influenced educators’ responses. However, our most recent work with ECE educators exploring the benefits of relational rewards shows results very similar to the current study, lending some degree of assurance for the current study findings. Also, as we have described, emerging conceptual work and reviews in the literature confirm the importance of workplace relationships to well-being.

We note that the disconnect educators described between themselves and upper administrators is likely more unique to large ECE systems with multiple administrative levels than to single-site early childcare and education programs in communities, limiting the generalizability of thematic findings. Finally, while enhancing the intrapersonal and interpersonal rewards that infant and toddler educators experience may be a beneficial strategy, it should not undermine the need to adequately compensate educators. According to the Early Childhood Workforce Index (Mclean et al., 2024), particularly infant and toddler educators, continue to be vastly underpaid with limited benefits. The report notes that early childhood educators are much more likely to need programs such as food assistance than their better-paid peers who are elementary or middle school classroom educators. Hence, the positive psychology strategies we have discussed must not distract from the necessity of fair compensation and positive working conditions for ECE educators. It is essential that society move forward to fully compensate educators for the complex work they do and their many contributions to a healthier society.

## Conclusions

Given the highly relational nature of early childhood programs, it is somewhat surprising that little work to date has focused on targeting intrapersonal and interpersonal relational stressors and rewards to enhance well-being and potentially reduce turnover. Our findings contribute to contemporary conceptual models of educator well-being (e.g., Jeon et al., 2026). Moreover, educators’ active use of intrapersonal and interpersonal positive experiences as intentional stress management strategies helps to elucidate known associations between workplace relationships and well-being. Further, we posit that models of well-being, including relational well-being, expand to reflect educators’ work as both stressful and rewarding and focus on their co-existence. We also suggest that models from other disciplines, including approaches grounded in positive psychology, specifically psychological rewards, and savoring approaches, can contribute to and extend ECE models of educator well-being.

## Figures and Tables

**Table 1:** Summary of themes

Theme 1: Stressful Experiences	Theme 2: Rewarding Experiences
**Intrapersonal Experiences**	
Cognitive & Emotional Load	Identity
*Balancing children’s developmental & emotional needs (planning how)*	*Work as a Calling & Valuing Impacts on Development*
**Interpersonal Experiences**	
Interactions	Interactions
*Balancing children’s needs (implementing practices)*	*Moments with children*
*Holding multiple roles in work with children & families*	*Benefitting from relationships with colleagues*
*Relationship experiences with administrators*	
*Feeling unseen/unheard*	
*Lack of understanding & support*	
*Poor communication*	
	**Theme 3: Intentional Efforts to Buffer Stress**
	Educators’ intentional reflections on identity & positive interactions to buffer stress & shield children from their stress

## Data Availability

Due to the small sample size and the specific identification of Early Head Start/Early Head Start Partnership programs, we cannot make full interview data available.
